# HLA-A29 Negative Birdshot-like Chorioretinopathy Associated with Vitiligo—Case Report

**DOI:** 10.3390/jcm13164808

**Published:** 2024-08-15

**Authors:** Julia Krzemińska, Anna Kurek, Agnieszka Żebrowska, Arleta Waszczykowska

**Affiliations:** 1 Department of Ophthalmology, Medical University of Lodz, 90-153 Lodz, Poland; julia.krzeminska1@gmail.com (J.K.); anna.katarzyna.kurek@gmail.com (A.K.); 2 Department of Dermatology and Venereology, Medical University of Lodz, 90-647 Lodz, Poland; agnieszka.zebrowska@umed.lodz.pl

**Keywords:** birdshot chorioretinopathy, choroidopathy, HLA-A-29, vitiliginous chorioretinitis, immunosuppressive treatment

## Abstract

A 54-year-old, one-eyed Caucasian male was admitted to the Ophthalmology Clinic due to a gradual deterioration of vision in the right eye for approximately two weeks. The patient denied any trauma or viral infection during this time. On the day of admission, the patient’s best corrected visual acuity (BCVA) in the right eye was 0.5 on the Snellen scale. The patient’s left eye had been atrophied for several years, with no light perception and no visibility of the fundus due to previous trauma and multiple surgeries. Ophthalmologic examination of the anterior segment and vitreous body of both eyes showed no signs of inflammation. Fundus examination of the right eye revealed scattered inflammatory foci, creamy-yellow and round, visible in all sectors. Laboratory tests, imaging studies, optical coherence tomography (OCT) angiographies, OCTs of the macula and optic nerve head, fluorescein angiographies (FAs), electroretinograms (ERGs), and visual field tests were performed. These examinations led to a diagnosis of a disease resembling birdshot-like chorioretinopathy. Immunogenetic testing of the patient did not reveal the presence of human leukocyte antigen (HLA)-A29. Dermatological and immunological consultations were conducted, and a differential diagnosis was made. Due to the reduced visual acuity (VA) observed and the inability to assess the left eye, a high-dose corticosteroid therapy was initiated, which was gradually tapered, along with the application of an immunosuppressive drug. The course of the disease was typical for birdshot chorioretinopathy, with chronic periods of remissions and exacerbations. The patient’s clinical improvement was only achieved after co-administration of general corticosteroids at a dose of 0.5–1 mg/kg/day, mycofenolate mofetil, and periocular (sub-Tenon’s) triamcinolone.

## 1. Introduction

Birdshot chorioretinopathy is a rare disease with a prevalence of less than 1 per 100,000 [1]. The disease was first described in 1980 by Ryan and Maumenee [2]. In most cases, birdshot chorioretinopathy is characterized by bilateral, multiple, oval or round, creamy-white choroidal lesions, which are 1/4–1/2 optic disc diameter in size [3,4]. These birdshot lesions are usually located around the optic disk, but may also appear peripherally [3]. The most common symptoms include blurred vision, floaters, nyctalopia, and dyschromatopsia. It is noteworthy that visual acuity (VA) often remains normal [4]. The disease affects almost exclusively adults, most often middle-aged Caucasians (the average age of onset is 53). The disease is diagnosed slightly more often in women [3,4]. The mechanisms involved in the pathogenesis of birdshot are incompletely understood [5]. A strong association between human leukocyte antigen (HLA)-A29 and the development of the disease has been reported. The presence of HLA-A29 is found in approximately 95.7% of patients [3,6]. However, according to the classification criteria for birdshot chorioretinitis developed by the Standardization of Uveitis Nomenclature (SUN) Working Group, a positive test result for HLA-A29 is not necessary for diagnosis [7]. There are no treatment guidelines for birdshot chorioretinopathy. So far, corticosteroids and immunosuppressive therapy remain the most commonly used medications [5]. The disease has a poor prognosis—its nature is chronic, progressive, and leads to vision loss [4,5]. However, according to several studies, treatment can slow the progression of the disease [8,9,10].

## 2. Case Report

A 54-year-old Caucasian male was admitted to the Ophthalmology Clinic due to a gradual deterioration of vision, decreased contrast sensitivity, and persistent floaters for approximately two weeks. The patient denied any recent trauma, viral infection, or other complaints. General medical history revealed gastro-oesophageal reflux on treatment with a proton pump inhibitor and an allergy to diclofenac.

While the history of the right eye was unremarkable, the left eye had a history of trauma approximately 40 years ago, with subsequent removal of a post-traumatic cataract, pars plana vitrectomy (PPV) with scleral buckling, injection of 1000 silicone oil, and a basal iridectomy (Ando). This was followed by uveitis and secondary glaucoma, leading to a loss of light perception in the left eye.

**Figure 1:**
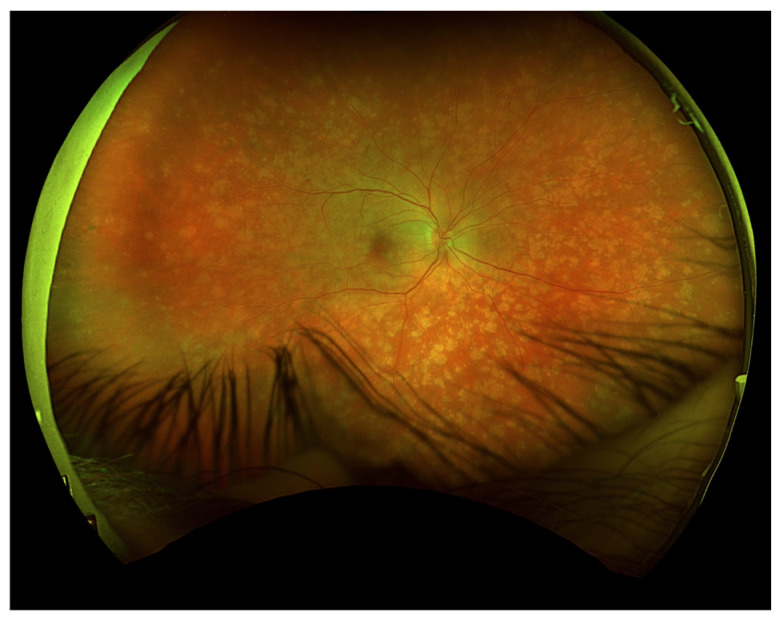
Fundus photography of the right eye depicted chorioretinal lesions in all quadrants.

Initial ophthalmic examination revealed a best corrected visual acuity (BCVA) of 0.5 on the Snellen scale in the right eye and no light perception in the left eye. Intraocular pressure (IOP) was 14.6 mmHg in the right eye and 31.8 mmHg in the left eye. Examination of the anterior segment of the right eye showed early lens opacities. No anterior chamber cells, keratic precipitates, posterior iris adhesions, or any other signs of anterior uveitis were revealed. Fundus examination of the right eye revealed multiple scattered, creamy-yellow, round chorioretinal lesions present in all sectors (Figure 1). The examination also noted 1+ vitreous cells, optic disc edema, and no signs of macular edema. A B-scan ultrasonography (USG-B) of the right eye showed posterior vitreous detachment and vitreous opacities.

Examination of the anterior segment of the left eye revealed conjunctival hyperemia, band keratopathy, and several focal corneal opacities. The iris examination showed rubeosis and a basal iridectomy at 6 o’clock. The pupil was decentered, irregular in shape, and non-reactive to light, with a pronounced post-inflammatory membrane. The posterior chamber intraocular lens was decentered inferiorly. Fundus examination of the left eye was not possible due to the lack of visibility. The USG-B result for the left eye was non-diagnostic due to the presence of silicone oil in the vitreous chamber. Similarly, other examinations described later in the article either could not be performed on the left eye or their results were non-diagnostic.

**Figure 2:**
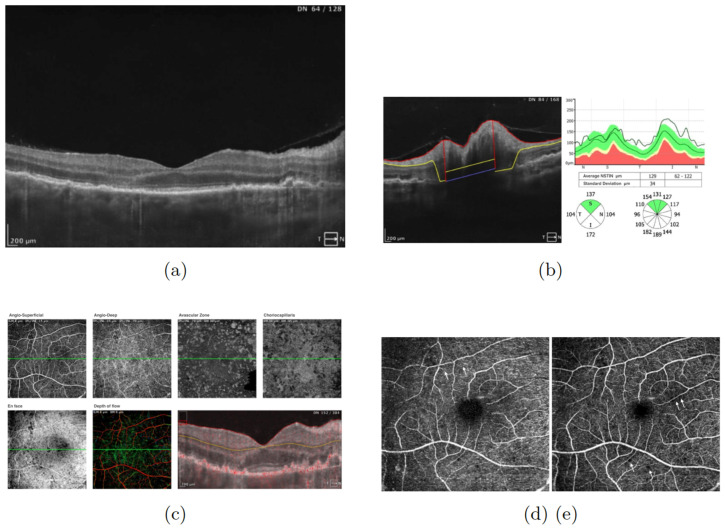
(**a**) Macular optical coherence tomography (OCT) of the right eye showed thickening of the outer nuclear layer (ONL) of the retina, drusen, and intraretinal cysts. Examination performed on the day of the patient’s hospital admission. (**b**) Retinal nerve fiber layer (RNFL) thickness graph showed increased thickness due to edema in the right eye. Examination performed on the day of the patient’s hospital admission. (**c**) OCT angiography showed capillary loops and increased intercapillary spaces as well as lesions visible in each vascular layer. Examination performed on the day of the patient’s hospital admission. (**d**) OCT angiography showed capillary loops (angio-superficial) (arrows). (**e**) OCT angiography showed increased intercapillary spaces (angio-deep) (arrows). Examination performed on the day of the patient’s hospital admission.

An optical coherence tomography (OCT) (SOCT REVO NX 130, Optopol Technology Sp. z o.o., Zawiercie, Poland) of the right eye was performed. The OCT of the macula revealed thickening of the outer nuclear layer (ONL) of the retina (Figure 2a), multiple disorganizations of the retinal pigment epithelium (RPE) (drusen), and intraretinal cysts. The OCT of the optic disc showed retinal nerve fiber layer (RNFL) thickness due to edema (thickening in temporal, nasal, and inferior quadrants) (Figure 2b). OCT angiography disclosed capillary loops and increased intercapillary spaces (reduced density in both the superficial and the deep plexus), which may be indicative of inflammation of the capillaries (Figure 2c–e). Moreover, the lesions were visible in each vascular layer (Figure 2c).

**Figure 3:**
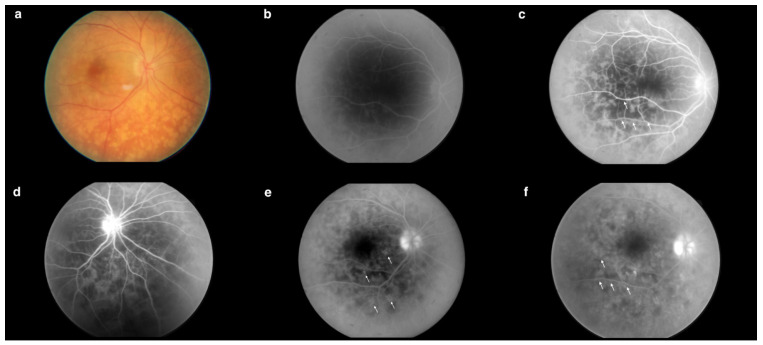
(**a**) Fundus photography of the right eye. (**b**) Increased arteriovenous transit time (0′27″). (**c**) Hypofluorescent choroidal lesions in early FA phases (arrows) (0′35″). (**d**) Intense optic nerve hyperfluorescence, which suggested optic disc edema (0′44″). (**e**) Increasing fluorescence over time occurred in only a few areas, indicating the presence of single leaks (arrows) (2′49″). (**f**) Hypofluorescent lesions in the early FA phases became hyperfluorescent in the late FA phases (arrows) (4′20″). Examination performed on the day of the patient’s hospital admission.

Fluorescein angiography (FA) (Figure 3b–e) of the right eye revealed an increased arteriovenous transit time of 27 s (Figure 3b). Moreover, the early phases of the FA showed hypofluorescent lesions (Figure 3c), which in later phases were replaced by multiple hyperfluorescent diffuse foci of high density (Figure 3f). Increasing fluorescence over time occurred in only a few areas, indicating the presence of single leaks (Figure 3e). The FA revealed blurred borders and intense optic disc hyperfluorescence (Figure 3d) visible from the early phases of the examination. In the posterior pole and the periphery, a spotty choroidal structure was visible throughout the examination (Figure 3a–f).

**Figure 4:**
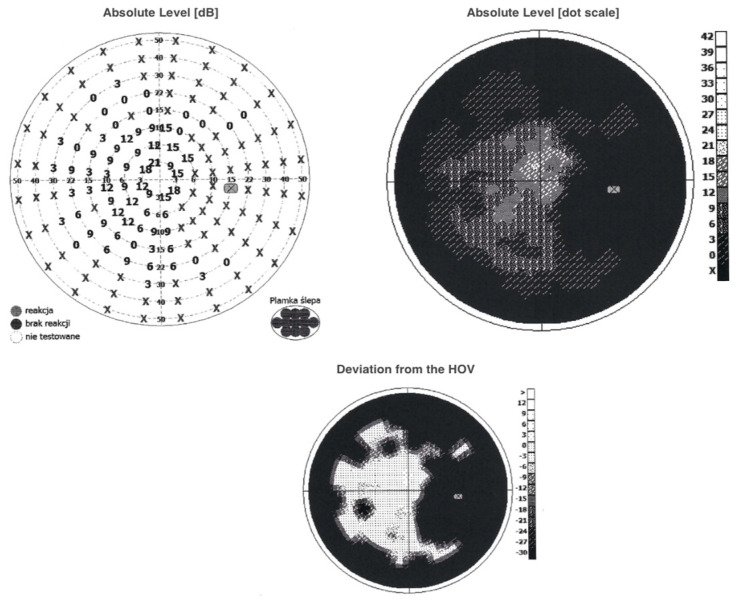
A visual field evaluation revealed large scotoma involving the blind spot and peripheral constrictions in all quadrants. Examination performed on the day of the patient’s hospital admission. HOV; hill of vision.

The visual field examination was notable for observed abnormalities in the central visual field—a large central scotoma involving the blind spot reaching 5 degrees from the fixation point. Peripheral constrictions in all quadrants were also present (Figure 4).

**Figure 5:**
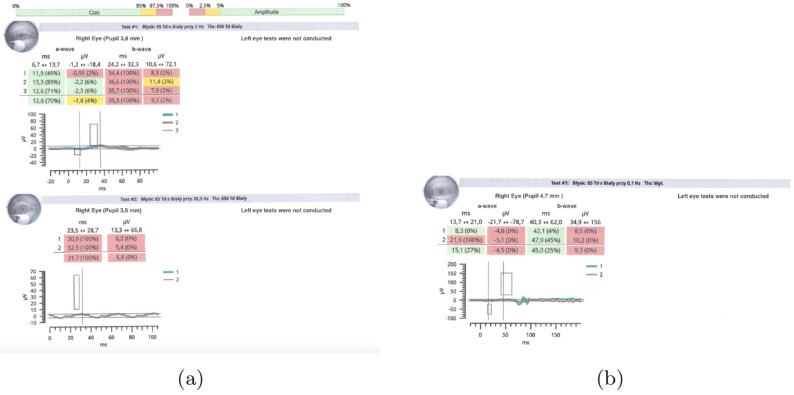
(**a**) Photopic flicker test. Examination performed on the day of the patient’s hospital admission. (**b**) Dark adaptation test. Examination performed on the day of the patient’s hospital admission.

An electroretinogram (ERG) test was conducted in a non-mydriatic pupil using the handheld RETeval system (LKC Technologies, Inc., Gaithersburg, MD, USA) with skin electrodes. The only eye examined was the right eye. Two testing protocols were used—a photopic flicker test (Figure 5a) and a scotopic test after a 20-min dark adaptation (Figure 5b). The flicker ERG revealed a reduction in amplitude and delayed latency (Figure 5a). However, the dark adaptation test showed a reduction in amplitude and latency in the reference range (Figure 5b).

Preliminary laboratory tests showed a slight decrease in eosinophil count and a slightly elevated urea level. Other laboratory assessments, including a complete blood count, an ionogram, and tests assessing coagulation parameters, kidney parameters, blood glucose, and C-reactive protein (CRP) were within normal limits. The laboratory tests performed for infectious etiologies are presented in Table 1. The result of the HLA-A29 test was negative. The chest X-ray did not show any abnormalities.

During the 9-day hospitalization, the patient received topical corticosteroids, a nonsteroidal anti-inflammatory drug, and 1% tropicamide for the right eye, as well as medications to lower intraocular pressure for the left eye and methylprednisolone administered intravenously at a dose of 40 mg per day.

Examination conducted at the point of hospital discharge showed a reduction in the severity of chorioretinal lesions. Visual field testing revealed a significant improvement relative to the previous examination. No other significant changes were observed compared to the admission examination.

On discharge from the hospital, the patient was prescribed topical corticosteroids, oral prednisone in decreasing doses—starting at 240 mg per day for 2 days, then at 120 mg for 5 days—and mycophenolate mofetil at a daily dose of 500 mg.

**Figure 6:**
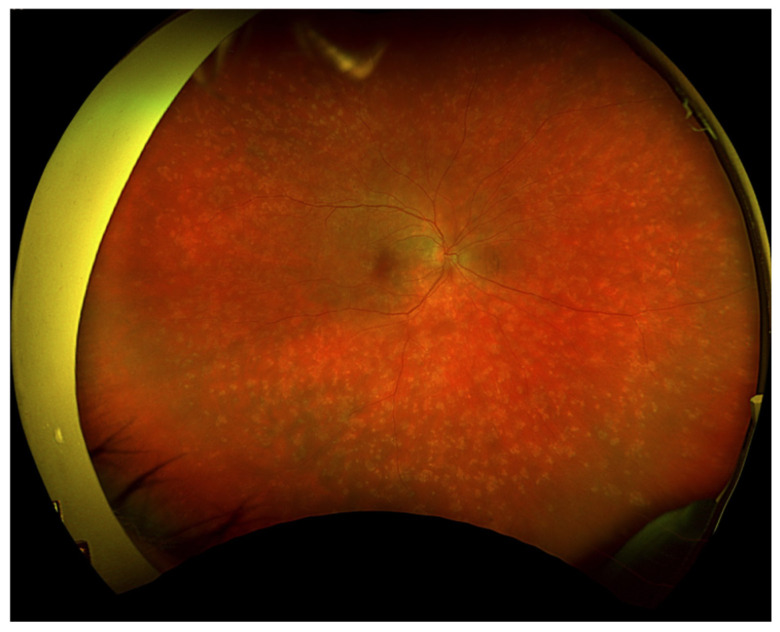
Fundus photography of the right eye depicted reduced chorioretinal lesions during the first follow-up visit (7 days after discharge from the hospital).

At the first follow-up, which took place one week after the hospitalization, the BCVA of the right eye was 0.5 (+2) on the Snellen scale. In the left eye there was still no light perception. The patient reported blurred vision and decreased contrast sensitivity; however, he recognized colors correctly. The IOP in the right eye was 12.2 mmHg, and in the left eye was 4 mmHg. The anterior segment examination of both eyes was as in the previous evaluations. On the fundus of the right eye, diffused, creamy-yellow, round inflammatory foci were visible in all quadrants. The lesions were smaller and significantly less noticeable compared to the previous examination (Figure 6). Tests performed included an ultrasound-B, as well as a visual field test, an OCT of the macula, an optic disc test, and an angio-OCT of the right eye.

An OCT of the macula in the right eye showed persistent retinal thickening, preserved foveal contour, and multiple drusen. An OCT of the optic disc showed edema, especially nasally and temporally.

OCT angiography also showed no significant differences from the previous study. Abnormalities such as capillary loops, focal dilatation, and increased interlobular space were seen in the study.

The visual field examination was notable for observed abnormalities in the central visual field—namely, a large central scotoma involving the blind spot. Peripheral constrictions in both upper quadrants and in the lower-nasal quadrant were also present.

Further treatment with topical dexamethasone and general treatment with mycophenolate mofetil 500 mg per day was maintained. The dose of prednisone was gradually reduced every 4 days, starting with 80 mg and in the end maintaining a 40 mg dose until the next follow-up visit. Subsequently, further follow-up has been scheduled and a date has been set for a brain magnetic resonance imaging (MRI) with contrast.

**Figure 7:**
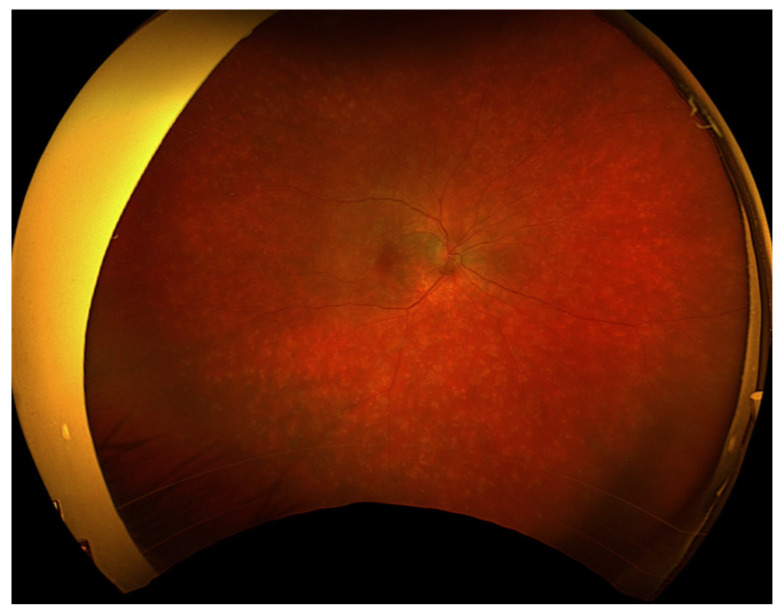
Regression of inflammatory lesions of the right eye fundus 14 days after discharge from the hospital.

**Figure 8:**
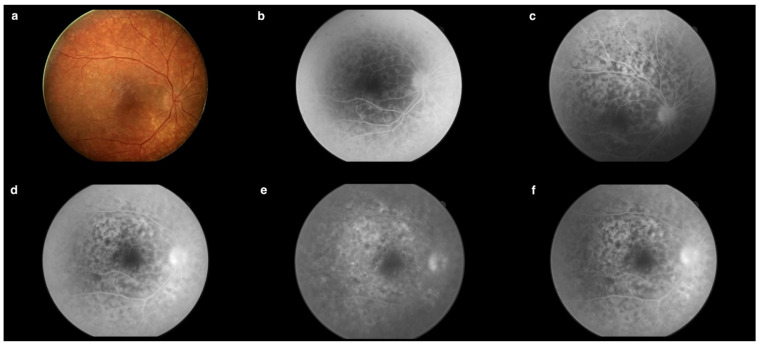
(**a**) Fundus photography of the right eye 14 days after discharge from the hospital. (**b**) Optic disc hyperfluorescence and edema were visible, persisting from the early phases of the examination (0′34″6). (**c**) Late phases of the leakage (0′48″6). (**d**) Leakage from large retinal vessels indicative of their inflammatory state and leakage from capillaries in the posterior pole was visible (1′22″4). (**e**) Increasing fluorescence over time demonstrated the presence of multiple leaks (3′23″0). (**f**) There was hyperfluorescence inferiorly and nasally within the foveal avascular zone (FAZ) (4′38″3).

At the next follow-up one week later (14 days after discharge from the hospital), a regression of the inflammatory lesions of the right eye fundus was observed (Figure 7 and Figure 8a). In the FA from the initial phases of this examination, there was a noticeable increase in the intensity of spotty choroidal structure throughout the posterior pole compared to the previous examination. The intensity of the lesions and fluorescence increased over time (Figure 8d,e). Leakage from large retinal vessels, indicative of their inflammation, and leakage from capillaries in the posterior pole were noticed (Figure 8d,e). Both optic disc hyperfluorescence and edema were visible, persisting from the early phases of the examination (Figure 8b,d). There was hyperfluorescence inferiorly and nasally within the foveal avascular zone (FAZ) (Figure 8f).

**Figure 9:**
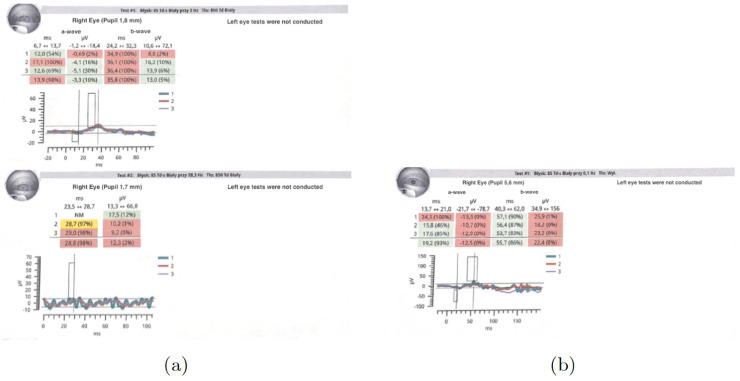
(**a**) ERG test—photopic flicker test performed 14 days after discharge from the hospital. (**b**) ERG test result—scotopic test performed 14 days after discharge from the hospital.

In the control ERG, the photopic flicker test (Figure 9a), a minimal reduction in latency and a more than twofold increase in amplitude (from 5.8 Hz to 12.3 Hz) was noted. In the scotopic test (Figure 9b), both alpha and beta waves revealed normal latency, and a slight reduction in amplitude was apparent. The above results indicated an improvement over the previous test. Our observation confirms the results of other authors who report that improvement in parameters in ERG testing may precede clinical symptoms of improvement [11].

MRI examination of the brain revealed a pineal cyst of 10 mm × 8 mm × 7 mm in size, as well as small mucosal thickening in the right maxillary sinus. In addition, the examination described an altered signal of the left eye due to the condition after left eye surgery (PPV with oil injection). The rest of the examination description was without deviations.

Four subsequent follow-up visits did not reveal significant changes. The ophthalmic examination was comparable to the examination performed during the second follow-up visit. Treatment was continued, with the corticosteroid dose being reduced from 40 mg by 4 mg every four days, from 34 mg to 28 mg, keeping the patient on this dose until the next, the seventh, follow-up visit.

**Figure 10:**
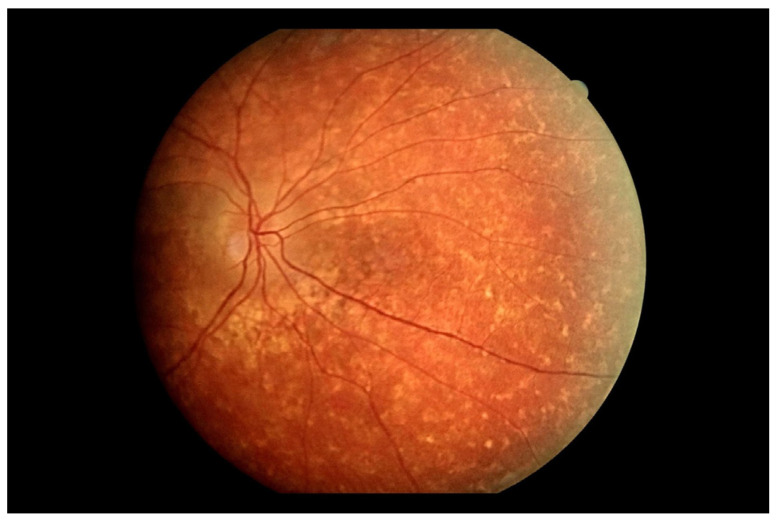
On the 76th day of treatment, fundus examination of the right eye revealed an increase in the severity of chorioretinal lesions.

At the next, seventh follow-up visit (76th day of treatment), the patient reported a deterioration in his condition. In recent days, his vision had worsened further, and he reported a significant decrease in contrast sensitivity. BCVA in the right eye was 0.32 on the Snellen scale. IOP in both eyes was 7.1 mmHg. Anterior segment examination showed a subconjunctival hemorrhage at 6 o’clock; besides this, the conjunctiva was unremarkable. The opacities in the vitreous body were visible on the USG-B scan of the right eye. The results of examination of the fundus remained similar to in previous evaluations (Figure 10), with further visible creamy-yellow lesions in all quadrants. Due to the patient’s worsening condition, it was decided to admit him to the hospital for the optimization of corticosteroid treatment in an inpatient setting.

The rehospitalization lasted 4 days. Laboratory tests performed during rehospitalization noted a decreased levels of platelets and eosinophilia, as well as lymphocytes and phosphates. In response to the increase in the level of phosphates, the level of parathormone was checked and was found to be within normal limits. The levels of immunoglobulins and white blood cells, including neutrophils, were shown to be above the normal limit. In addition, elevated levels of total cholesterol and triglycerides, ALAT, and urea were reported. A general urine examination showed no abnormalities.

Upon admission, OCTs of the macula and optic disc as well as an angio-OCT were performed; however, the results were not different from those previously performed. The OCT of the optic disc was non-diagnostic due to numerous opacities. In the following days, the patient underwent regular ophthalmic examinations. The assessment of the patient’s condition showed even greater deterioration, as the BCVA of the right eye was 0.2 on the Snellen scale.

**Figure 11:**
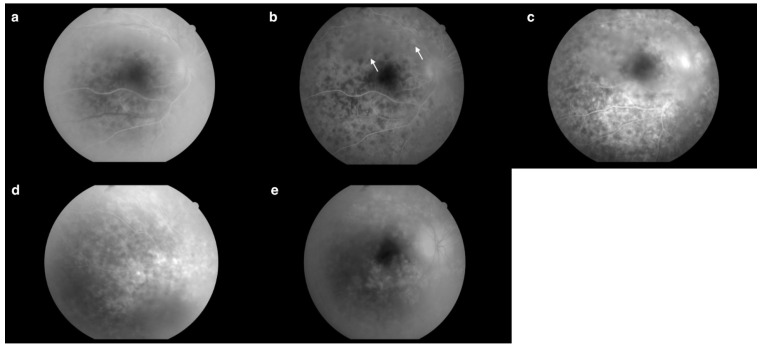
(**a**) Optic disc hyperfluorescence and edema persisting from the early phases of the examination (0′15″). (**b**) Visible dense opacities in the vitreous body obscuring the upper temporal arcade of vessels and the optic disc with, moreover, visible leakage (arrows) (0′28″). (**c**) Visible opacities in the vitreous body, hyperfluorescence and edema of the optic disc, as well as hyperfluorescence inferiorly and nasally within the FAZ (1′04″). (**d**) Inflammatory lesions and leaks visible around the periphery of the retina (2′06″). (**e**) Massive leaks localized mainly around the optic disc and the macula (5′39″). Examination performed during rehospitalization on the 76th day of treatment.

In the FA, macular choroidal structure was visible in each phase of the examination. In addition, an increase in fluorescence over time was noticeable during the examination. Hyperfluorescence and edema of the optic disc both persisted from the early phases of the examination (Figure 11a). A prolonged arteriovenous transit time was also observed. As seen in Figure 11b, dense opacities in the vitreous body obscured the upper temporal vascular arcade and the optic nerve disc. Moreover, leaks were visible. During the entire examination, opacities in the vitreous body and hyperfluorescence and edema of the optic nerve disc prevail, as well as hyperfluorescence inferiorly and nasally within the FAZ (Figure 11c). Inflammatory lesions and leaks were visible around the periphery of the retina (Figure 11d). In the late phases of the examination, massive leaks were visible, localizing mainly around the optic disc and the macula. A heavily blurred image was seen due to the presence of opacities in the vitreous body (Figure 11e).

During rehospitalization, general treatment with prednisone at 120 mg per day was administered. Both mycophenolate mofetil at a dose of 500 mg per day and a topical treatment with dexamethasone were maintained.

Due to the patient’s deteriorating condition he qualified for periocular (sub-Tenon’s) triamcinolone administration to the right eye.

For further treatment at home, prednisone at a dose of 100 mg and acetazolamide at a dose of 500 mg were prescribed. Topical corticosteroidal treatment was continued with the addition of an antibiotic due to the injection.

The patient attended a follow-up visit 4 days after rehospitalization (84th day of treatment) to evaluate IOP after the corticosteroid dose was increased. Ocular evaluation showed gradual improvement in the vision of the right eye and BCVA was 0.4 (+1). The IOP of the right eye was 10.2 mmHg. The left eye remained unchanged. Therefore, the corticosteroid dose was maintained awaiting further improvement, and both the acetazolamide and the IOP-lowering medications were discontinued.

**Figure 12:**
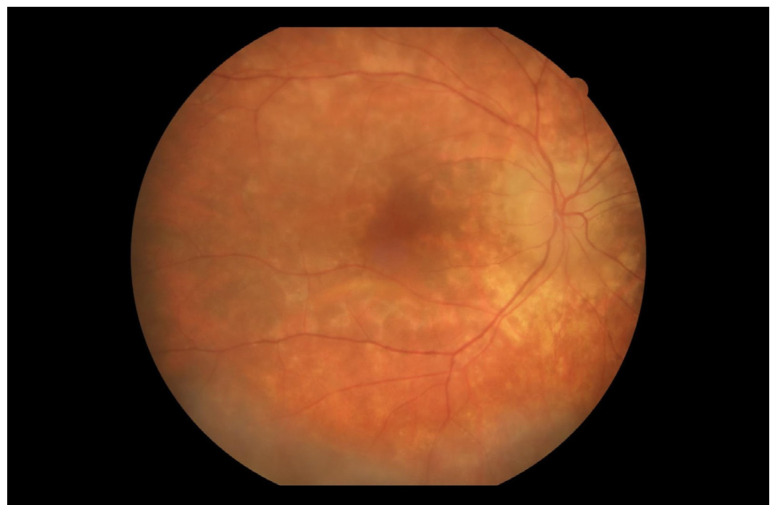
Fundus photography of the right eye showed a reduction in the severity of chorioretinal lesions. Examination performed on the 98th day of treatment.

Two weeks after the triamcinolone administration (98th day of treatment), the patient attended a follow-up appointment. The patient reported great improvement compared to previous examinations. Ocular evaluation disclosed a BCVA of 0.7 in the right eye and no light perception in the left eye. IOPs were 20.6 mmHg and 7.1 mmHg, respectively. The result of the anterior segment examination of both eyes was as in the previous evaluations. Fundus examination of the right eye revealed a great reduction in the severity of the chorioretinal lesions in all quadrants (Figure 12). The optic disc edema was still evident. The left eye fundus remained unexaminable. A USG B-scan of the right eye showed a reduction of opacities in the vitreous body compared to the examination.

**Figure 13:**
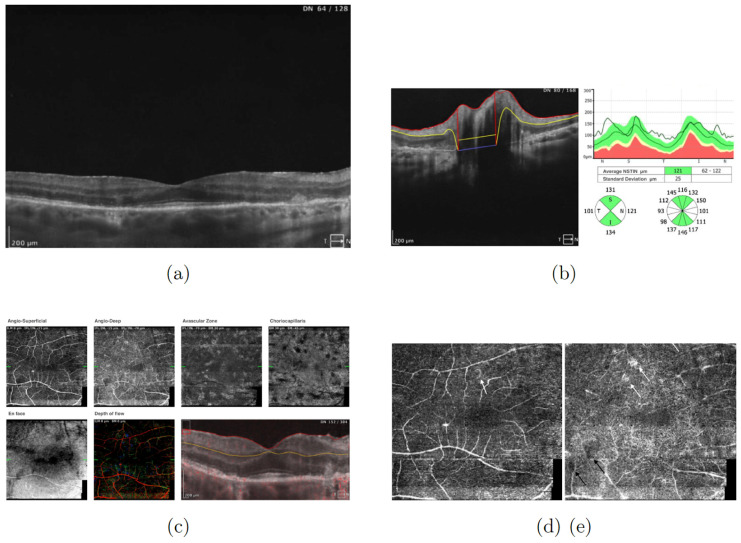
(**a**) OCT of the macula showed thickening of the ONL of the retina. Examination performed on the 98th day of treatment. (**b**) RNFL thickness graph showed thickening in temporal and nasal quadrants. Examination performed on the 98th day of treatment. (**c**) OCT angiography showed lesions present in each vascular layer. Examination performed on the 98th day of treatment. (**d**) OCT angiography showed capillary loops (angio-superficial) (arrows). (**e**) OCT angiography showed increased intercapillary spaces (black arrows) and capillary loops (white arrows) (angio-deep). Examination performed on the 98th day of treatment.

An OCT of the macula of the right eye showed persistent retinal thickening, with disorganization of the RPE and deposit material in the ONL located nasally from the foveola; apart from that, the RPE was normal (Figure 13a). OCT of the right eye optic disc showed RNFL thickness due to edema (thickening in the temporal and nasal quadrants) (Figure 13b).

OCT angiography results were similar to in previous examinations—capillary loops and reduced density in both the superficial and the deep plexus were visible (Figure 13c–e). In addition, the lesions present in each vascular layer persisted (Figure 13c).

**Figure 14:**
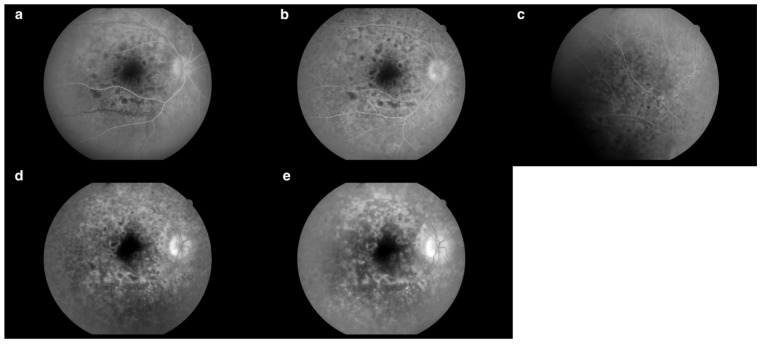
(**a**) Hypofluorescent choroidal lesions in early phases. Blurred borders of the optic disc (0′07″). (**b**) Spotty choroidal structure in the posterior pole (0′20″). (**c**) Spotty choroidal structure with lower intensity at the periphery (0′25″). (**d**) Hyperfluorescence inferiorly and nasally within the FAZ. Choroidal lesions surrounded by hyperfluorescence (1′22″). (**e**) Hyperfluorescence inferiorly and nasally within the FAZ. Intense hyperfluorescence of the optic disc. Leakages from small vessels (4′50″). Examination performed on the 98th day of treatment.

The FA showed significant improvement compared to previous examinations. The regression of the lesions from the periphery was evident as a spotty choroidal structure with lower intensity at the periphery (Figure 14c), occurring mainly in the posterior pole (Figure 14b). Each choroidal lesion in the early phases was hypofluorescent (Figure 14a), while in the later phases each lesion was surrounded by hyperfluorescence (Figure 14d). The fovea remained unchanged; however, there was apparent hyperfluorescence inferiorly and nasally within the FAZ, similar to previous examinations (Figure 14d,e). The FA revealed blurred borders of the optic disc visible from the early phases of the examination (Figure 14a). However, intense hyperfluorescence did not appear until later phases (Figure 14e). Leaks from small vessels were visible, possibly indicative of their inflammation (Figure 14e).

The pharmacotherapy was modified at this point—the prednisone dose was gradually reduced from 100 mg per day to 60 mg per day. Both topical corticosteroids and oral mycophenolate mofetil at a daily dose of 500 mg were maintained.

**Figure 15:**
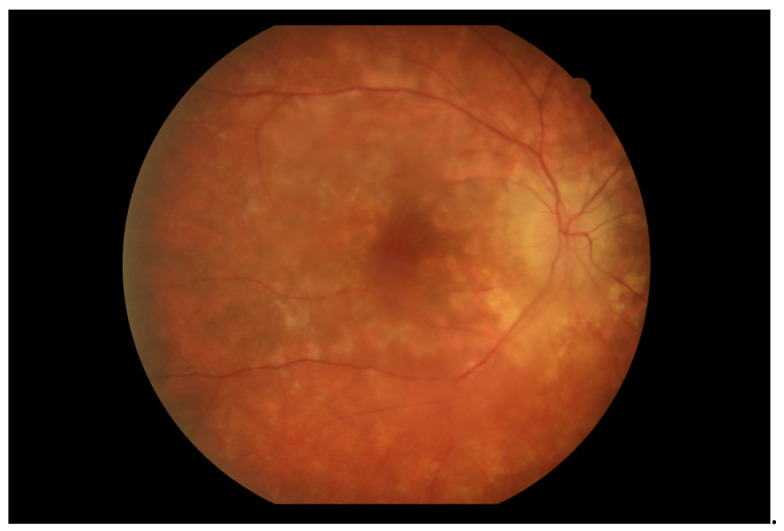
Fundus photography of the right eye. Examination performed on the 118th day of treatment.

Over a month after the administration of triamcinolone (118th day of treatment),another follow-up visit was held. According to the patient’s report, a condition similar to the previous examination persisted. Ophthalmologic evaluation showed a BCVA of 0.7 in the right eye and no light perception in the left eye. The IOPs were 14.6 mmHg and 7.1 mmHg, respectively. Examination of the anterior segment of the eye was the same as in previous evaluations. Examination of the fundus of the right eye showed no major changes compared to the examination at the previous follow-up (Figure 15). Edema of the optic nerve disc was still evident. The fundus of the left eye remained unexaminable. B-scan ultrasound of the right eye showed slightly increased opacity in the vitreous body compared to the previous examination. The results of the OCT examination of the macula and the optic disc, as well as the FA of the right eye, did not differ from the previous inspection.

The pharmacotherapy was again modified. The dose of prednisone was reduced from a daily dose of 60 mg to 55 mg. The remaining general and topical treatments were maintained.

## 3. Discussion

Birdshot chorioretinopathy is a chronic, progressive disease primarily affecting middle-aged adults [3]. The etiology of the disease remains unknown; however, the presence of HLA-A29 is found in nearly 96% of patients [6]. The Genome Wide Association Study (GWAS) showed that increased expression of Endoplasmic reticulum aminopeptidase (ERAP) 2 with coexisting decreased expression of ERAP1 can activate an immune response in the choroid of HLA-A29 carriers [12]. The role of T lymphocytes in the etiology of birdshot is also presumed—they are the most frequently detected cells on histopathological examination [13]; moreover, studies have shown the presence of CD4+ and CD8+ lymphocytes in the vitreous body [14].

While HLA-A29 is relatively common in the European population (7–9%), very few HLA-A29 positive individuals develop birdshot, suggesting that HLA-A29 is insufficient to explain the pathophysiology of the disease, and so its presence has not been considered a required criterion for diagnosis. The presence of the HLA-A29 antigen only poses a risk of disease [5].

Birdshot chorioretinopathy is strongly associated with the HLA-A29 antigen, which is an important diagnostic marker for the disease. However, there are rare cases in which patients with clinical symptoms of birdshot are negative for HLA-A29. Coussa et al. [15] described cases of patients with symptoms typical for a birdshot chorioretinopathy with negative HLA-A29. There were clinical cases of individuals aged 22 and 13 who were diagnosed with common variable immunodeficiency (CVID). During routine ophthalmologic examinations, reduced VA, bilateral optic disc edema, and choroidal-retinal infiltration in the form of cream-colored lesions without features of vitritis were found on fundus evaluation. The result of the HLA-A29 determination was negative. After diagnostics, no infectious, inflammatory, or neoplastic etiologies were identified. Other causes of similar symptoms were ruled out. De Maeyer et al. [16] described the case of a 17-year-old girl with asymptomatic bilateral optic disc edema and retinal infiltrates. These findings were noted during routine follow-up visits for hydroxychloroquine use for lymphoid interstitial pneumonia. The patient described by de Maeyer et al. [16] was diagnosed with CVID with splenomegaly, lymphadenopathy, and lymphoid interstitial pneumonia at the age of 13. On examination, VA was 6/5 in both eyes. Anterior segment examination was unremarkable, while a fundoscopy revealed bilateral edema of the optic disc and chorioretinal infiltrates resembling bird’s arrows, with no signs of vitreous inflammation. An MRI of the brain showed no signs of increased intracranial pressure. HLA typing was negative for HLA-A29. De la Fuente et al. [17] also described an example of a patient with both fundus lesions typical for birdshot and a negative HLA-A29 antigen. The retinal inflammatory lesions involved one eye, making the description even more similar to the patient we discussed. It is important to consider and exclude other substrates of uveitis, which may manifest similar symptoms but differ in therapeutic approach and prognosis.

During our observation, both the severity of symptoms and the VA varied. On admission, our patient presented the most common symptoms of birdshot chorioretinopathy, which are blurred vision (88%) and floaters (43%). Moreover, he reported a characteristic decreased contrast sensitivity [3]. His BCVA was 0.5, while at the most severe point of deterioration the BCVA dropped to 0.2 and symptoms such as floaters significantly increased. During more recent observations, the patient’s condition improved significantly. His BCVA reached 0.7, and the severity of the symptoms decreased significantly. The variability of symptoms over time, along with the periods of remission and exacerbation, showed the recurrent character of the disease.

Researchers emphasize the special role of FAs in the diagnosis of birdshot chorioretinopathy [5]. The lesions are typically hypofluorescent in the early phases and demonstrate hyperfluorescence in the late phases. Both hyperfluorescence of the optic nerve disc and prolonged arteriovenous transit time—which, according to clinical studies, averages 31.1 ± 5.2 s in birdshot patients—are also common symptoms [18,19]. We observed all these features in the case described. The FA results correlated with the patient’s clinical condition, as indicated by the withdrawal of peripheral lesions at the time of the last follow-up, when the greatest improvement was documented. Studies suggest that the ERG is a sensitive tool for monitoring changes in patients with birdshot [11]. In the observation of our clinical case, we noted an improvement in all assessed ERG parameters after the treatment. Furthermore, to our knowledge, this is only the second study describing the use of the ERG RETeval system for monitoring birdshot chorioretinopathy [20].

Due to the lack of visibility into the fundus of the left eye, it was impossible to perform many of the tests helpful in diagnosing birdshot. As a result, we had no way of knowing whether the lesions were present unilaterally or bilaterally. Four cases of patients with unilateral lesions characteristic for birdshot chorioretinopathy have been described so far [17,21,22,23].

De la Fuente et al. [17] presented a case of an HLA-A29 negative patient diagnosed with unilateral birdshot-like choroidopathy. The patient presented with unilateral fundus lesions in the form of multiple round hypopigmented lesions in the choroid. The choroidal lesions were also visible in the FA and the indocyanine green angiography (ICGA). Although observed for 30 months, the lesions did not appear in the other eye. Another case of unilateral birdshot was described by Akiki et al. [23]. The HLA-A29 positive patient presented fundus lesions characteristic for birdshot, which were also visible on ICGA in one eye only. The character of the lesions did not differ from the previous study, which took place 3 years earlier. A similar case is the HLA-A29 positive patient reported by Marrero et al. [22], whose examination revealed features characteristic for birdshot chorioretinopathy, such as hypopigmented lesions and retinal vasculitis that occurred unilaterally. In contrast, there was an HLA-A29 positive patient described by Huang et al. [21] whose lesions characteristic for birdshot chorioretinopathy first appeared unilaterally and then, after about a year, developed in the other eye as well. This may suggest that the disease may start asynchronously. According to the research criteria developed by the international consensus conference [24], to which many of the case reports described in the literature refer, the presence of the disease in both eyes is among the required characteristics. However, according to the classification criteria for birdshot chorioretinitis developed by the SUN Working Group [7], it can be diagnosed in certain cases even when the lesions occur unilaterally. Undoubtedly, birdshot chorioretinopathy should also be considered when the characteristic lesions are unilateral.

Although a positive test result for HLA-A29 and the presence of lesions bilaterally are not necessary for the diagnosis, the unfortunate facts that we could not diagnose lesions bilaterally and that the patient was HLA-A29 negative made it impossible to meet the classification criteria for birdshot choroidopathy developed by the SUN Working Group in our patient [7].

Since there are no treatment guidelines, Bousquet et al. [5] developed, based on the available literature, a summary for the treatment of birdshot chorioretinopathy. According to the proposed regimen, the treatment started with high doses of general corticosteroids and immunosuppressive therapy. Despite the absence of cystic macular edema, due to the blindness of the other eye we decided to prescribe high doses of prednisone and mycophenolate mofetil, with a concomitant use of topical corticosteroids. For about two months after hospitalization, the patient’s condition was stable and the lesions on the fundus seemed to be decreasing, so the prednisone dose was gradually reduced. Unfortunately, an exacerbation of the disease occurred at the dose of 28 mg per day. The BCVA then reached the lowest value we recorded during the entire follow-up period of the patient, and floaters increased significantly. At that point, the corticosteroid dose was increased to 120 mg per day and subtenon triamcinolone was administered. This resulted in an improvement—the BCVA then reached the highest value we recorded during the entire patient observation. Our patient’s case suggests that in some patients doses of general corticosteroids should be maintained in the range of 0.5–1 mg/kg/day for a longer period of time despite a stable condition, and that additional administration of triamcinolone subtenon is an effective method of controlling the disease.

In the course of differential diagnosis using laboratory and imaging examinations, we excluded syphilis and sarcoidosis as exclusion criteria for the diagnosis of birdshot. Considering the results of the brain MRI, blood tests, and the outcome of the immunological consultation, the suspicion of intraocular lymphoma was removed. Due to the fact that it was a one-eyed patient, an invasive diagnostics (vitreous biopsy) was not opted for. Tuberculosis, Borrelia burgdorferi, Toxoplasma gondii, CMV and HIV infections were also excluded. Since our patient presents an atypical picture of the disease, the differential diagnosis was large-scaled. Based on the clinical picture, sympathetic inflammation, Vogt–Koyanagy–Harada syndrome (VKH), and other white dot syndromes were all excluded. Multiple Evanescent White Dot Syndrome (MEWDS) and Acute Posterior Multifocal Placoid Pigment Epitheliopathy (APMPPE) were excluded mainly due to the self-limiting nature of these diseases [25], as well as their regression without treatment within a few weeks and rare recurrences [26,27]. The exclusion of MEWDS and APMPPE was also suggested by the nature of the lesions. In these diseases, the lesions are located mainly in the posterior pole [25,27], which did not correspond to the fundus image of the described patient. Furthermore, a history of recent viral infection, although its not obligatory, did not occur in this case. In addition, early hyperfluorescence is characteristic for MEWDS on FA [26], whereas early hypofluorescence and late hyperfluorescence were observed. The suspicion of Multifocal Choroiditis and Panuveitis (MCP) was dismissed based on the absence of anterior uveitis throughout the follow-up. In addition, the nature of the fundus lesions differs—in MCP the inflammatory foci merge [28], whereas in the patient’s case the lesions’ borders were well-defined.

In the dermatological examination, a vitiligo lesion was visible on the back at the level of the right scapula. According to the patient, this lesion had been present on the skin for several years. There were no similar lesions in other locations. The remaining skin and mucous membranes showed no pathological lesions. In the collected medical history, there were no symptoms of connective tissue disease or other autoinflammatory dermatoses. Blood was drawn for immunological tests, which revealed antinuclear antibodies at a titer of 1:160 with a speckled pattern (considered an insignificant result). In the ANA3 panel, the following were not detected: nRNP/Sm, Sm, SS-A, SS-B, Ro-52, Scl-70, Jo-1, DFS70, ribosomal P protein, centromeres, dsDNA, or PM-SCL antibodies. Anti-CCP antibodies were absent, and the rheumatoid factor was negative.

On immunological evaluation, a number of abnormalities were revealed. Laboratory tests were notable for lymphopenia of 880 cells/µL with neutrophilia of 6720/µL, platelets at the lower limit of normal, non-significantly decreased IgG1, and slightly increased IgG4. IgD was below the reference range. A flow cytometry study showed a reduction in the number of NK cells and an increased CD4/CD8 ratio, while the absolute values of the aforementioned subpopulations’ counts were normal. Moreover, the analysis showed that the C2 component of complement was at the lower limit of normal. Other complement components are within reference range. Elevated levels of specific IgEs indicate that the patient was allergic to grass pollen. Most importantly regarding birdshot chorioretinopathy was the absence of deviations in the levels of immunoglobulins A, G, M and E, excluding CVID, which is prevalent in HLA-A29 negative patients [16].

During the dermatological consultation, a diagnosis of vitiligo was made in the described patient. This is important because vitiligo, as an autoimmune disease, can coexist with birdshot-type retinal inflammatory lesions. They pay particular attention to the possible role of melanocyte-derived antigens, noting reports of an association with vitiligo and other skin diseases and that patients with birdshot chorioretinopathy have a higher than expected rate of skin (and other) cancers [1,18].

Gass et al. [18] described the similarity in appearance and evolution of vitiliginous chorioretinitis patches to those found on the skin of patients with vitiligo. Fouad et al. [29] demonstrated that vitiligo patients had lower subfoveal choroidal thickness compared to healthy individuals, indicating that vitiligo affects the choroid. Furthermore, vitiligo is more common in people with uveitis than in the general population. LeWitt et al. [30] noted the correlation of vitiligo with changes in the retina and choroid. They described a number of changes that occurred on the fundus in patients with vitiligo, ranging from characteristic choroidal changes called ‘tigroid fundus’ to multiple retinal pigmentary abnormalities. Ocular manifestations of vitiligo include a range of diseases such as VKH and Alezzandrini syndromes, but also include tear film abnormalities, dry eye syndrome, and ocular surface diseases. In addition, Minos et al. [1] demonstrated the similarity to areas of skin depigmentation observed in vitiligo. Hesse et al. [31] described a case of a birdshot chorioretinopathy patient with long-standing typical psoriasis. These references, along with the aforementioned case of birdshot retinopathy, suggest a possible strong connection between birdshot chorioretinopathy and immunodermatological disorders such as vitiligo and psoriasis.

## 4. Conclusions

We presented a clinical case of a patient with an atypical clinical manifestation of birdshot-type chorioretinopathy. The patient presented many features typical for birdshot, such as the fundus image, the results of additional tests, primarily the FA and ERG. Moreover, the course of the disease was typical for birdshot—chronic with periods of remissions and exacerbations. Therapy applied according to the regimen proposed for birdshot treatment resulted in improvement. However, due to the lack of visibility into the fundus of the left eye, it was not known whether the lesions were present unilaterally or bilaterally. Moreover, the result of the HLA-A29 test was negative. As a result, it was impossible to meet the classification criteria for birdshot choroiditis developed by the SUN Working Group.

This case illustrates that birdshot chorioretinopathy can present atypically. Even if patients do not meet the standard classification criteria, extensive diagnostics are necessary. This includes laboratory tests, additional diagnostic tests, specialist consultations, and differential diagnosis. The case demonstrated that in some instances, therapeutic benefit can only be achieved when a multi-drug therapy (general and topical corticosteroids, mycophenolate mofetil, and sub-Tenon’s triamcinolone) is applied over many months. Furthermore, in some patients it may not be possible to reduce doses of general corticosteroids to <0.5 mg/kg/day for several months, as it otherwise leads to deterioration. The case is also a confirmation of the efficacy of periocular (sub-Tenon’s) triamcinolone administration, which should be considered in patients whose clinical condition necessitates high-dose immunosuppressive therapy. A limitation of the study is the duration of the follow-up, which lasted approximately 4 months. Long-term follow-up is planned.

## Figures and Tables

**Table 1:** Laboratory evaluations for infectious etiologies.

Laboratory Test	Results
anti-HIV 1 and anti-HIV 2 antibodies	negative
antigen HIV 1	negative
anti-CMV IgM antibodies	negative
anti-CMV IgG antibodies	positive
anti-Borrelia burgdorferi IgM antibodies	negative
anti-Borrelia burgdorferi IgG antibodies	negative
anti-Toxoplasma gondii IgM antibodies	negative
anti-Toxoplasma gondii IgG antibodies	negative
QuantiFERON-TB	negative
FTA-ABS	negative
WR (anti-Treponema pallidum antibodies)	non-reactive
ACE	in reference range
IgE sp. nGal d 4 (lysozyme)	negative

HIV, human immunodeficiency virus; CMV, cytomegalovirus; FTA-ABS, fluorescent treponemal antibody absorption; WR, Wassermann reaction; ACE, angiotensin converting enzyme.

## Data Availability

The data used in this article are sourced from materials mentioned in the References section and the medical records of the Department of Ophthalmology, Medical University of Lodz, Poland.
